# Deubiquitinase UCHL1 regulates estradiol synthesis by stabilizing voltage-dependent anion channel 2

**DOI:** 10.1016/j.jbc.2023.105316

**Published:** 2023-10-04

**Authors:** Shengjie Shi, Guiyan Chu, Lutong Zhang, Huan Yuan, Mielie Madaniyati, Xiaoge Zhou, Liguang Wang, Chuanjiang Cai, Weijun Pang, Lei Gao, Gongshe Yang

**Affiliations:** 1 College of Animal Science and Technology, Northwest A&F University, Yangling, China; 2 Key Laboratory of Animal Genetics, Breeding and Reproduction of Shaanxi Province, Yangling, China

**Keywords:** deubiquitination, estradiol, granulosa cells, UCHL1, VDAC2

## Abstract

Lack of estradiol production by granulosa cells blocks follicle development, causes failure of estrous initiation, and results in an inability to ovulate. The ubiquitin-proteasome system plays a critical role in maintaining protein homeostasis and stability of the estrous cycle, but knowledge of deubiquitination enzyme function in estradiol synthesis is limited. Here, we observe that the deubiquitinase ubiquitin C-terminal hydrolase 1 (UCHL1) is more significant in estrous sows and high litter-size sows than in nonestrous sows and low-yielding sows. Overexpression of UCHL1 promotes estradiol synthesis in granulosa cells, and interference with UCHL1 has the opposite effect. UCHL1 binds, deubiquitinates, and stabilizes voltage-dependent anion channel 2 (VDAC2), promoting the synthesis of the estradiol precursor pregnenolone. Cysteine 90 (C90) of UCHL1 is necessary for its deubiquitination activity, and Lys45 and Lys64 in VDAC2 are essential for its ubiquitination and degradation. *In vivo*, compared with WT and sh-NC-AAV groups, the estrus cycle of female mice is disturbed, estradiol level is decreased, and the number of antral follicles is decreased after the injection of sh-UCHL1-AAV into ovarian tissue. These findings suggest that UCHL1 promotes estradiol synthesis by stabilizing VDAC2 and identify UCHL1 as a candidate gene affecting reproductive performance.

Estradiol (E_2_) produced by granulosa cells (GCs) is essential for follicle development (1). E_2_ is responsible for enhancing the activity of mitosis-related kinases in GCs, promotes GC proliferation, maintains the survival of GCs and oocytes by inhibiting the levels of pro-apoptotic genes, prevents follicle degeneration due to GC apoptosis (follicular atresia), and promotes the formation of dominant follicles (2, 3). Insufficient secretion of E_2_ arrests follicular development in primary and secondary follicles before the formation of the follicular cavity (pre-antral follicular stage); E_2_ levels in follicular fluid positively correlate with follicle size and quality (4). In preovulatory follicles, large amounts of E_2_ feedback to the hypothalamus, causing spikes in follicle-stimulating hormone and luteinizing hormone, leading to ovulation. E_2_ metabolism is indispensable in the reproductive system and is a critical regulator of energy and glucose homeostasis affecting several biological activities (5). The estrogen receptor is expressed throughout the body. E_2_ binds the estrogen receptor to regulate physiological processes in breast tissue (6), bone (7), and the cardiovascular system (5). E_2_ is synthesized in GCs by several enzymatic reactions using cholesterol as a substrate, regulated by precise expression of many enzymes and proteins (8), including steroidogenic acute regulatory protein (StAR, the cholesterol transporter to the mitochondrial inner membrane), CYP11A1 (which converts cholesterol to pregnenolone referred to as PREG), 3β-HSD (which converts PREG to progesterone), and CYP19A1 (aromatase).

The ubiquitin-proteasome system degrades misfolded or damaged proteins; it also regulates protein levels, stability, and activity to maintain the balance of physiological states such as cell cycle, signal transduction, and cell metabolism (9). Ubiquitination is a highly regulated and reversible process. Deubiquitinating enzymes (DUBs) balance ubiquitination modifications and are expressed in several cell types (10). In mammals, E3 ubiquitin ligases and DUBs play central roles in protein degradation and turnover through protein ubiquitination and deubiquitination (11). Bebington and Doherty found that ubiquitin-related proteins may be involved in gametocytogenesis, placental development, and endometrial modifications at the onset of pregnancy and regulating steroid receptor concentrations (12). Nevertheless, the role of ubiquitination in E_2_ synthesis is unclear. Ubiquitin C-terminal hydrolase 1 (UCHL1, also known as PARK5/PGP9.5) is a DUB responsible for removing ubiquitin or polyubiquitin from target proteins (13). Previous studies showed that UCHL1 plays an essential role in tumorigenesis and metastasis (14) and the nervous (15) and reproductive systems in oocyte maturation (16), spermatogenesis (17), and preventing polyspermy (18). One study reported that UCHL1 is a candidate gene for influencing ovulation numbers in sows (19) and another demonstrated the importance of UCHL1 in ovarian development in mice (20). So we hypothesized that UCHL1 might play a role in E_2_ synthesis and thus affect follicle development. Nevertheless, the role of UCHL1 in E_2_ synthesis remains unclear.

Therefore, the present study used mass spectrometry–based deep ubiquitinome and interactome analysis to identify UCHL1 substrates. We identified previously unknown putative UCHL1 substrates and revealed many cellular pathways highly associated with UCHL1, particularly in steroid hormone synthesis. We also found that the level of UCHL1 in ovarian tissue of estrus sows is higher than that of nonestrus sows, and UCHL1 positively regulates E_2_ synthesis. We demonstrated that voltage-dependent anion channel 2 (VDAC2), a mitochondrial membrane protein responsible for cholesterol metabolism, is a specific substrate of UCHL1. UCHL1-mediated deubiquitination and stabilization of VDAC2 promote cholesterol transport to the mitochondrial inner membrane, revealing a previously unknown mechanism of E_2_ synthesis in GCs.

## Results

### Expression pattern of UCHL1 in porcine ovary

**Figure 1:**
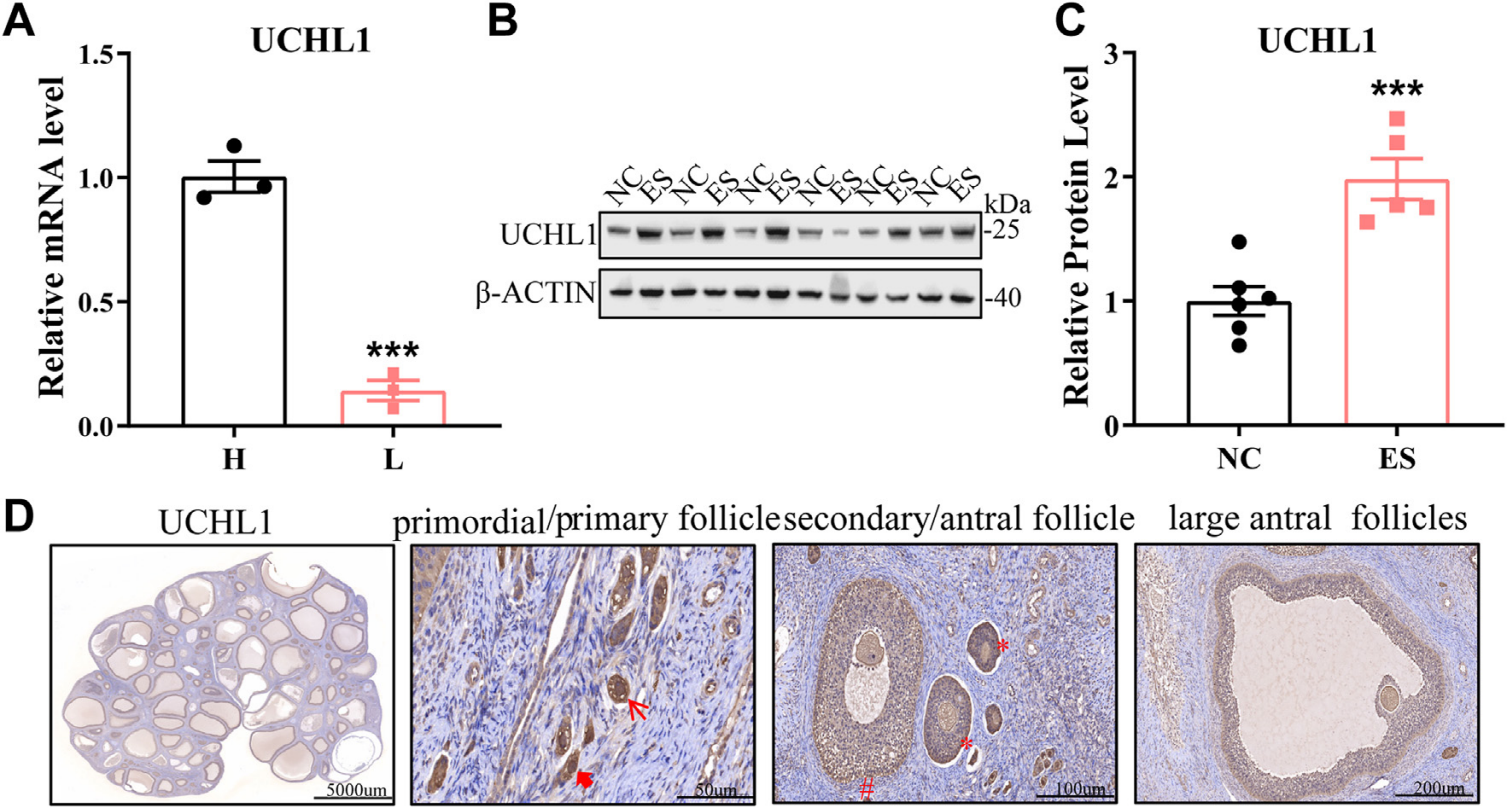
**Analysis of expression characteristics of UCHL1.***A*, mRNA levels of UCHL1 in ovarian tissues of high- and low-litter sows. *B*, UCHL1 protein levels in ovarian tissues of nonestrous and estrous sows. *C*, quantification of the Western blot analysis. *D*, immunohistochemical results reveal the localization of UCHL1 in the porcine ovary. Data are means ± SEMs of three independent experiments; ∗∗∗*p* < 0.001. UCHL1, ubiquitin C-terminal hydrolase L1.

To determine the expression pattern of UCHL1 in the porcine ovary, we measured UCHL1 levels in the ovarian tissues of high- and low-litter Yorkshire × Landrace sows. UCHL1 levels in the ovarian tissue of high-yielding sows were much higher than that of low-yielding sows (Fig. 1*A*). UCHL1 was more highly expressed in the ovarian tissue of estrous sows than that of nonestrous sows (Fig. 1, *B* and *C*). The expression pattern of UCHL1 in follicles at various stages was observed using immunohistochemistry of porcine ovarian tissue. UCHL1 was highly expressed in follicular GCs at various stages (Fig. 1*D*). These findings may suggest that UCHL1 positively regulates follicular development.

### UCHL1 promotes E_2_ synthesis in porcine GCs

**Figure 2:**
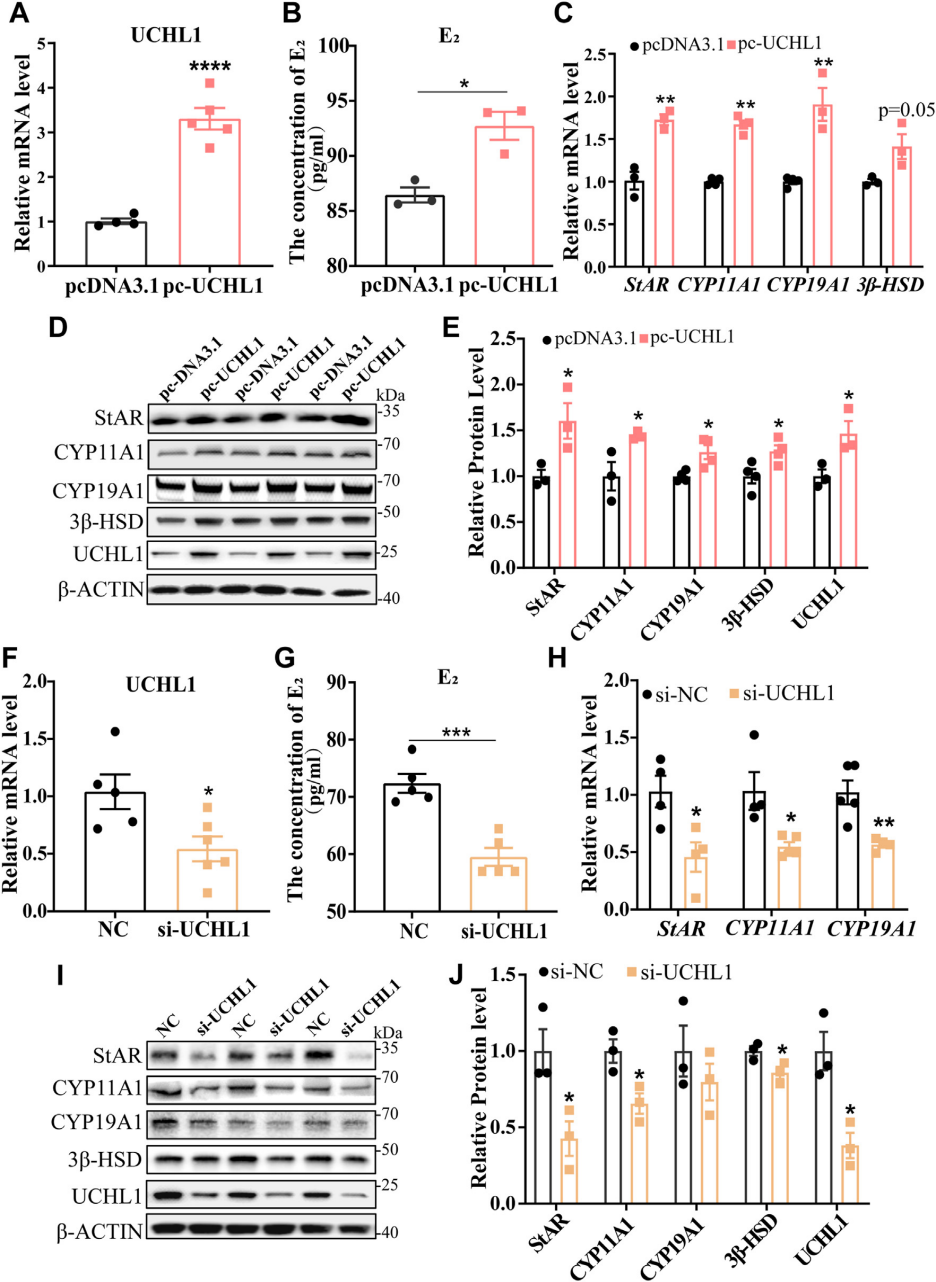
**UCHL1 promotes E**_**2**_**synthesis in GCs.***A*, overexpression efficiency of UCHL1 after transfection with pcDNA3.1-UCHL1 compared to pcDNA3.1. *B*, E_2_ levels were measured using ELISA. Culture supernatants were collected 24 h after pcDNA3.1-UCHL1 and pcDNA3.1 treatments. *C* and *H*, RT-qPCR detected critical genes in E_2_ synthesis, including StAR, CYP11A1, 3β-HSD, and CYP19A1. *D* and *I*, Western blot analysis of critical proteins in E_2_ synthesis. *E* and *J*, quantification of the Western blot analysis. *F*, inhibitory efficiency after transfection with si-UCHL1 compared with NC. *G*, E_2_ concentrations were measured using ELISA. Culture supernatants were collected 24 h after si-UCHL1 and NC treatments. Data are means ± SEMs of three independent experiments; ∗*p* < 0.05, ∗∗*p* < 0.01, ∗∗∗*p* < 0.001, ∗∗∗∗*p* < 0.0001. GC, granulosa cell; UCHL1, ubiquitin C-terminal hydrolase L1.

To evaluate the effects of UCHL1 on E_2_ synthesis, GCs were transfected with pcDNA3.1 and pcDNA3.1-UCHL1. UCHL1 mRNA level increased approximately three-fold (Fig. 2*A*). Notably, the overexpression of UCHL1 promoted increases in concentrations of E_2_ (Fig. 2*B*) and upregulated mRNA (Fig. 2*C*) and protein (Fig. 2, *D* and *E*) expression of steroid hormone synthetic markers including StAR, CYP11A1, CYP19A1, and 3β-HSD. In contrast, interference with UCHL1 produced effects opposite to those of overexpressing UCHL1 (Fig. 2, *F*–*J*).

We also examined the role of UCHL1 inhibitor LDN57444 on E_2_ synthesis. Consistent with our expectations, after LDN57444 treatment, UCHL1 enzymatic activity was inhibited (Fig. S1*A*), the concentrations of E_2_ in the medium decreased (Fig. S1*B*), and the protein levels of steroid hormone synthesis–related markers decreased (Fig. S1, *C* and *D*). These results suggest that UCHL1 plays a positive role in E_2_ synthesis.

### Annotation of UCHL1-regulated ubiquitinome and proteome

**Figure 3:**
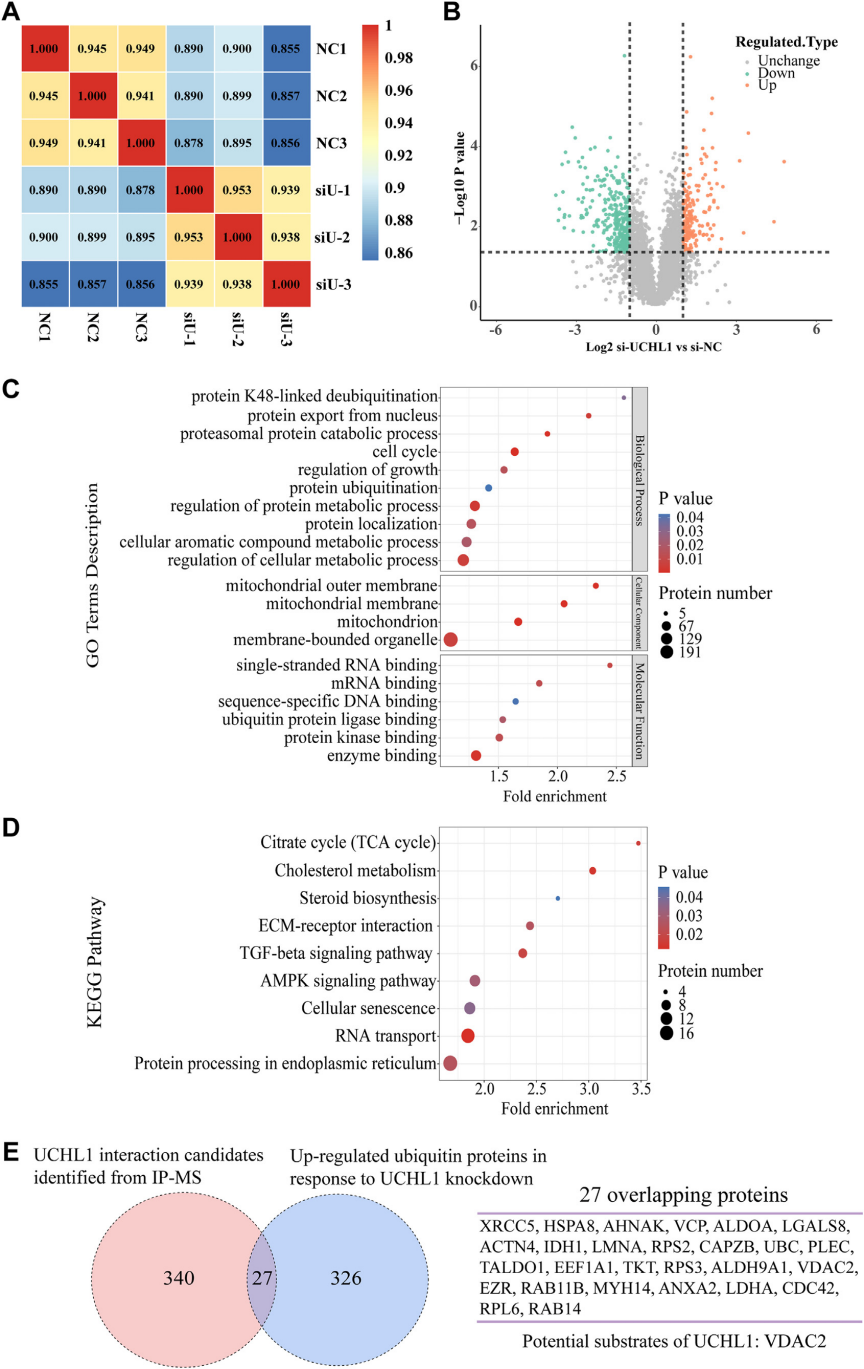
**Ubiquitinome and proteome profiling for UCHL1-regulated proteins and interaction components.***A*, heat map by calculating Pearson’s correlation coefficient between all pairs of samples. *B*, volcano plot of the protein abundance changes in response to si-UCHL1. The average protein expression ratio of three replicates (log2-transformed) between UCHL1 knockdown and NC porcine GCs was plotted against *p*-value by *t* test (-log 10 transformed). *C*, GO analysis of the significantly upregulated ubiquitin sites for biological process, cellular component, and molecular function. *D*, KEGG pathways of the significantly upregulated ubiquitin sites. *E*, the Venn diagram shows the number of upregulated ubiquitin sites identified in response to si-UCHL1, UCHL1 interaction candidates identified in the Co-IP process, and overlapping proteins in the datasets. The chart below shows the potential substrates of the overlapped proteins. Co-IP, coimmunoprecipitation; GC, granulosa cell; GO, gene ontology; UCHL1, ubiquitin C-terminal hydrolase L1.

To identify the substrates of UCHL1, we performed quantitative proteomics of ubiquitination modification, an affinity-based ubiquitinated peptide enrichment approach to quantify the change of ubiquitinome in si-UCHL1 GCs systematically. First, we demonstrated the statistical consistency of the samples by calculating the Pearson correlation coefficient between the pairings (Fig. 3*A*). Using a criterion of ≥ 1.5-fold change between the groups, the ubiquitization sites of 353 proteins were significantly upregulated (*p* < 0.05 by the Student’s *t* test) (Data S1), which may be regulated by knocking down UCHL1 (Fig. 3*B*). Based on the MoMo analysis results, we used a heat map to show the variation degree of amino acid occurrence frequency near the modification site to check whether there was any amino acid preference adjacent to the ubiquitinated sites. Alanine and aspartic acid were notably overrepresented close to the ubiquitin sites (Fig. S2*A*).

To gain insights into the biological functions in which UCHL1 may be involved, bioinformatics analysis of gene ontology and Kyoto Encyclopedia of Genes and Genomes and their domains of UCHL1-upregulated proteins were performed. The results revealed that UCHL1 might be involved in cholesterol metabolism and steroid biosynthesis signaling pathways (Fig. 3*D*), and its domains are also enriched in steroid-binding domains (Fig. S2*B*). Furthermore, our analysis revealed that UCHL1 might participate in cellular biological processes such as proteasome-dependent degradation, ubiquitin-protein ligand binding, mitochondrial function, and the cell cycle.

To determine the direct binding substrate of UCHL1 in porcine GCs, we performed immunoprecipitation-mass spectrometry (IP-MS) to investigate the interacting partners of UCHL1. UCHL1 pull-down samples of GCs were digested with trypsin and analyzed using MS. We obtained 367 UCHL1 potential binding proteins (Data S2). To determine the direct substrates of UCHL1, we compared the proteins whose ubiquitinated sites were upregulated and interacting proteins from the immunoprecipitation experiment and found proteins identified from two datasets. VDAC2 has attracted our attention because it participates in cholesterol transport associated with E_2_ synthesis (21).

### Verification of VDAC2 as a UCHL1 deubiquitination target in porcine GCs

**Figure 4:**
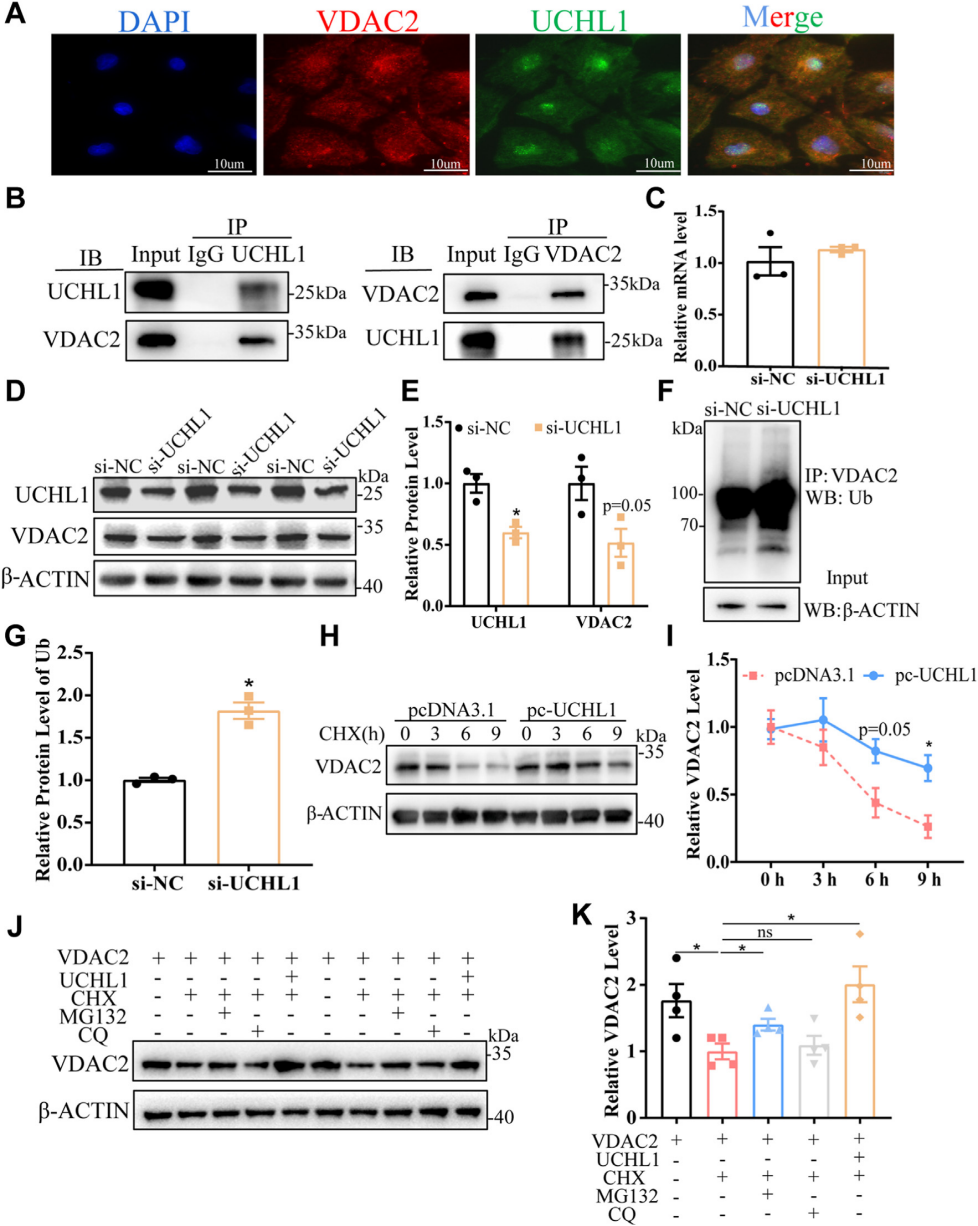
**Identification of VDAC2 as a direct target of UCHL1.***A*, representative immunofluorescence staining of UCHL1 and VDAC2 localization in GCs. *B*, Co-IP assay revealed an interaction between UCHL1 and VDAC2 using anti-UCHL1 or anti-VDAC2. *C*, relative mRNA levels of VDAC2 in GC transfection with si-UCHL1 for 24 h. *D*, protein levels of UCHL1 and VDAC2 were detected after GCs transfection with si-UCHL1 for 24 h. *E*, quantitative protein levels of UCHL1 and VDAC2. *F*, protein extracts were harvested after GC cells were transfected with si-UCHL1 or scrambled siRNA. Protein extracts were immunoprecipitated using an anti-VDAC2 antibody and analyzed with a Western blot using an anti-ubiquitin antibody. *G*, quantification of the Ub protein level. *H*, GCs with or without UCHL1 overexpression were treated with 25 μg/ml cycloheximide (CHX) for the indicated times. The whole cell lysate was assayed using western blots. *I*, the quantitative protein level of VDAC2 under (*H*) treatment. *J*, GCs were transfected with pcDNA3.1-VDAC2 and treated with CHX (25 μg/ml) alone or with MG132 (10 μM), CQ (50 μM) as a combination for 6 h, or transfected with the UCHL1 plasmid. The cell lysates were analyzed using western blotting. *K*, quantitative protein levels of VDAC2 under (*J*) treatment. Data are means ± SEMs of three independent experiments; ∗*p* < 0.05. Co-IP, coimmunoprecipitation; GC, granulosa cell; UCHL1, ubiquitin C-terminal hydrolase L2; VDAC2, voltage-dependent anion channel 2.

VDAC2 is a potential target of UCHL1 identified by ubiquitinome and IP-MS and is responsible for cholesterol transport to the mitochondrial inner membrane. However, it is necessary to demonstrate that VDAC2 is the target protein directly deubiquitinated by UCHL1. Confocal imaging showed that VDAC2 colocated with UCHL1 in GCs (Fig. 4*A*). We also performed a coimmunoprecipitation (Co-IP) assay to determine whether UCHL1 associates with VDAC2. We found that VDAC2 was precipitated by an antibody against UCHL1 but not by control immunoglobulin G in GCs. Reverse Co-IP confirmed that UCHL1 was precipitated by an antibody against VDAC2 (Fig. 4*B*). Interference with UCHL1 resulted in decreased VDAC2 protein (Fig. 4, *D* and *E*) but not its mRNA levels in GCs (Fig. 4*C*) due to enhanced poly-ubiquitination of VDAC2 (Fig. 4, *F* and *G*). These findings suggest that UCHL1 regulates VDAC2 expression through deubiquitination at the posttranslational level.

We measured the half-life of VDAC2 in GCs treated with the protein synthesis inhibitor cycloheximide (CHX). UCHL1 overexpression significantly prolonged the half-life of VDAC2 compared with the control group (Fig. 4, *H* and *I*). To study the stabilizing effect of UCHL1 on VDAC2 caused by its deubiquitination, we examined the protein stability of VDAC2 in the presence of CHX. We observed that the degradation of VDAC2 was rescued by a proteasome inhibitor (MG132) and UCHL1 overexpression but not by an autophagolysosome inhibitor (CQ) (Fig. 4, *J* and *K*). These findings suggest that UCHL1 stabilizes VDAC2 protein levels by deubiquitination.

### VDAC2 enhances cholesterol transport to the mitochondrial inner membrane

**Figure 5:**
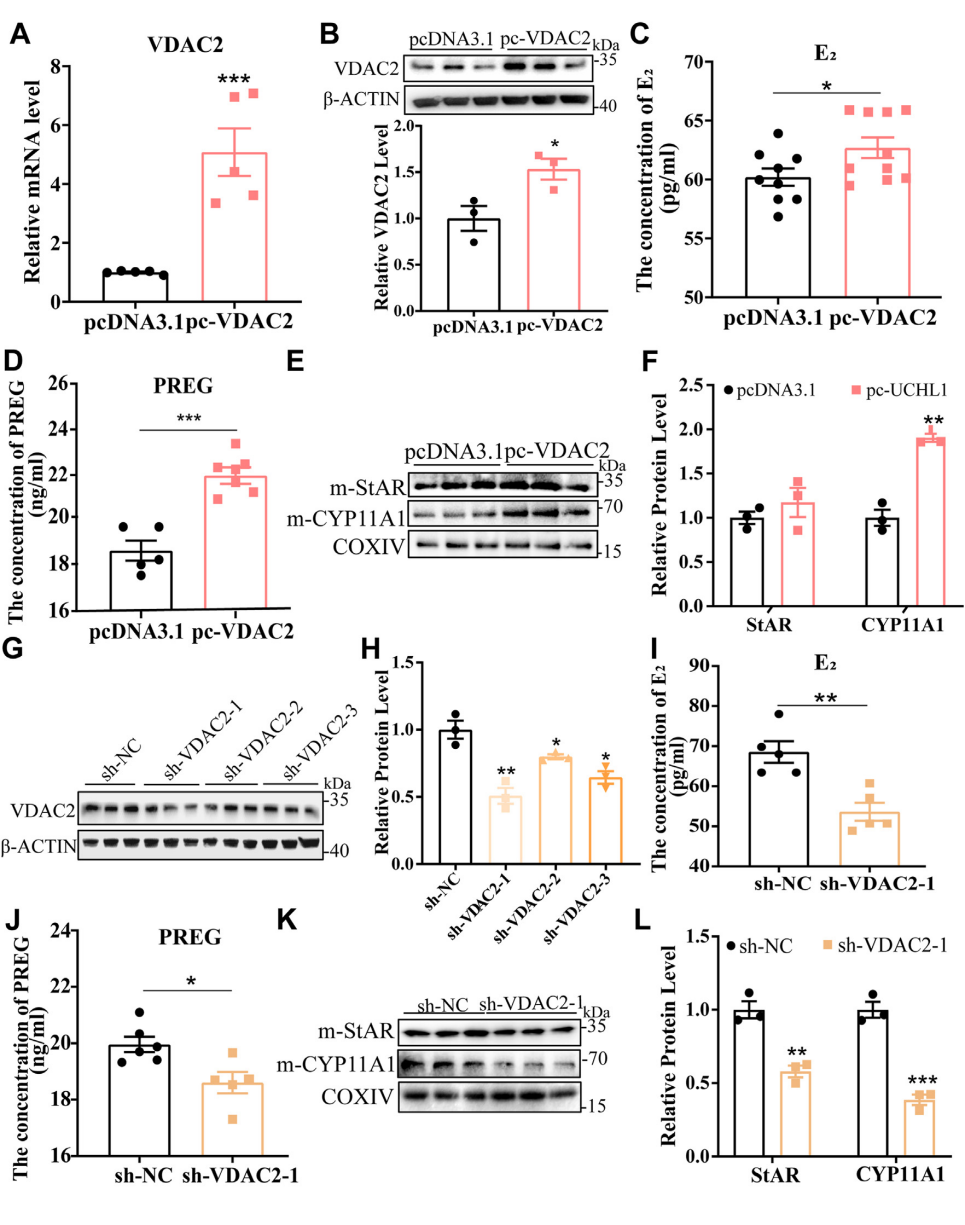
**VDAC2 promotes the synthesis of E**_**2**_**and PREG in porcine GCs.***A* and *B*, overexpression efficiency of VDAC2 after transfection with pcDNA3.1-VDAC2 compared to pcDNA3.1 at mRNA (*A*) and protein levels (*B*). *C* and *D*, E_2_ (*C*) and PREG (*D*) concentrations were measured using ELISA. Culture supernatants were collected 24 h after pcDNA3.1-VDAC2 and pcDNA3.1 treatments. *E*, protein levels of StAR and CYP11A1 in GC mitochondria after pcDNA3.1-VDAC2 and pcDNA3.1 treatments. *F*, quantitative protein levels of (*E*). *G*, the protein levels of VDAC2 in different sh-VDAC2 treatments compared with sh-NC. *H*, quantitative protein levels of (*G*). *I* and *J*, E_2_ (*I*) and PREG (*J*) concentrations were measured using ELISA. Culture supernatants were collected 24 h after sh-VDAC2-1 and sh-NC treatments. *K*, protein levels of StAR and CYP11A1 in GC mitochondria after sh-VDAC2 and sh-NC treatments. *L*, quantitative protein levels of (K). Data are means ± SEMs of three independent experiments; ∗*p* < 0.05, ∗∗*p* < 0.01, ∗∗∗*p* < 0.001. GC, granulosa cell; VDAC2, voltage-dependent anion channel 2.

Cholesterol is the substrate for steroid hormone synthesis. As a mitochondrial protein, VDAC2 controls the entry of StAR into mitochondria and affects the transport process of cholesterol to the inner mitochondrial membrane (21). As expected, after the overexpression of VDAC2 at the mRNA (Fig. 5*A*) and protein levels (Fig. 5*B*), E_2_ concentration was elevated (Fig. 5*C*). After cholesterol is transported to the inner mitochondrial membrane, it is converted into PREG by CYP11A1. Overexpression of VDAC2 elevated the concentration of PREG in the GC medium (Fig. 5*D*) by boosting the protein levels of StAR and CYP11A1 in the GC mitochondria (Fig. 5, *E* and *F*). In contrast, we constructed VDAC2-shRNA to knock down VDAC2 and found that sh-VDAC2-1 had the most significant knockdown efficiency (Fig. 5, *G* and *H*); this vector was used for subsequent tests. Consistent with the previous results, the concentrations of E_2_ (Fig. 5*I*) and PREG (Fig. 5*J*) in the sh-VDAC2 group were significantly lowered, and StAR and CYP11A1 in mitochondria were also lower than in the control group (Fig. 5, *K* and *L*). These findings suggest that VDAC2 promotes E_2_ synthesis by enhancing cholesterol transport to the mitochondrial inner membrane.

### VDAC2 mediated the promoting effect of UCHL1 on E_2_ synthesis in GCs

**Figure 6:**
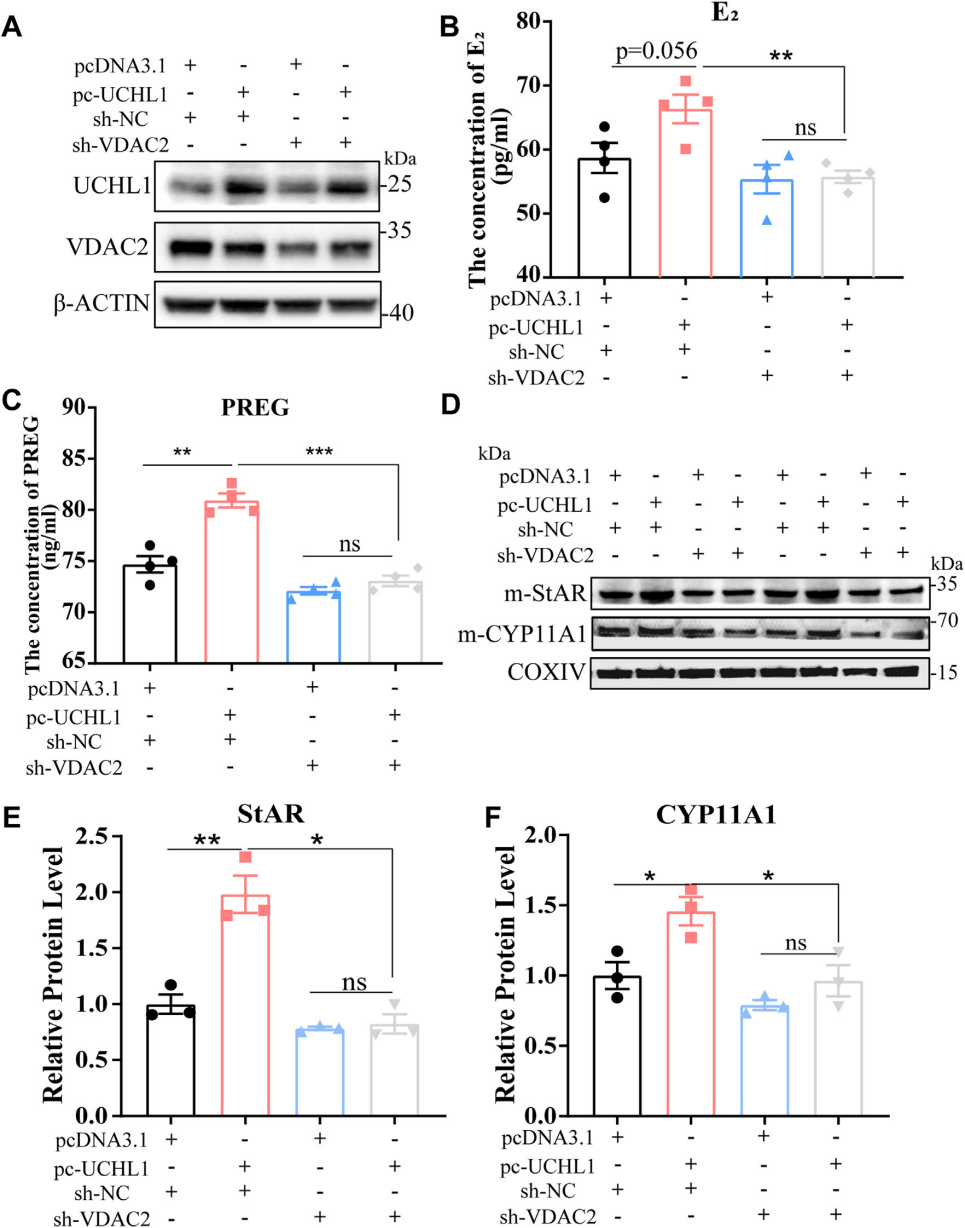
**VDAC2 mediated the promoting effect of UCHL1 on E**_**2**_**synthesis in GCs.***A*, representative protein levels of UCHL1 and VDAC2 after pc-UCHL1 cotransfection with sh-VDAC2. *B* and *C*, E_2_ (*B*) and PREG (*C*) concentrations were measured using ELISA. Culture supernatants were collected 24 h after pc-UCHL1 cotransfection with sh-VDAC2. *D*, protein levels of StAR and CYP11A1 in GC mitochondria in different treatments. *E* and *F*, quantitative protein levels of StAR (*E*) and CYP11A1 (*F*) under (*D*) treatments. Data are means ± SEMs of three independent experiments; ∗*p* < 0.05, ∗∗*p* < 0.01, ∗∗∗*p* < 0.001. GC, granulosa cell; UCHL1, ubiquitin C-terminal hydrolase L1; VDAC2, voltage-dependent anion channel 2.

Our previous findings suggested that UCHL1 regulates the stability of VDAC2 through its deubiquitination effect, and VDAC2 promotes cholesterol transport to mitochondria. These findings prompted us to investigate whether VDAC2 mediates the effect of UCHL1 on E_2_ synthesis. We performed shRNA-mediated VDAC2 knockdown with UCHL1 overexpression in GCs (Fig. 6*A*). VDAC2 knockdown largely blocked the roles of UCHL1 overexpression (Fig. 6, *B* and *C*), suggesting that the role of UCHL1 in the regulation of E_2_ synthesis, at least in part, depends on the upregulation of VDAC2.

Considering the role of VDAC2 in cholesterol transport, we also extracted mitochondrial proteins in different treatment groups. We found that sh-VDAC2 cotransfected with pc-UCHL1 reduced the expression levels of StAR and CYP11A1 compared with the group of pc-UCHL1 (Fig. 6, *D*–*F*). These findings suggest that the role of UCHL1 in E_2_ synthesis depends on regulating the deubiquitination of VDAC2.

### C90 of UCHL1 is necessary for its deubiquitination activity

**Figure 7:**
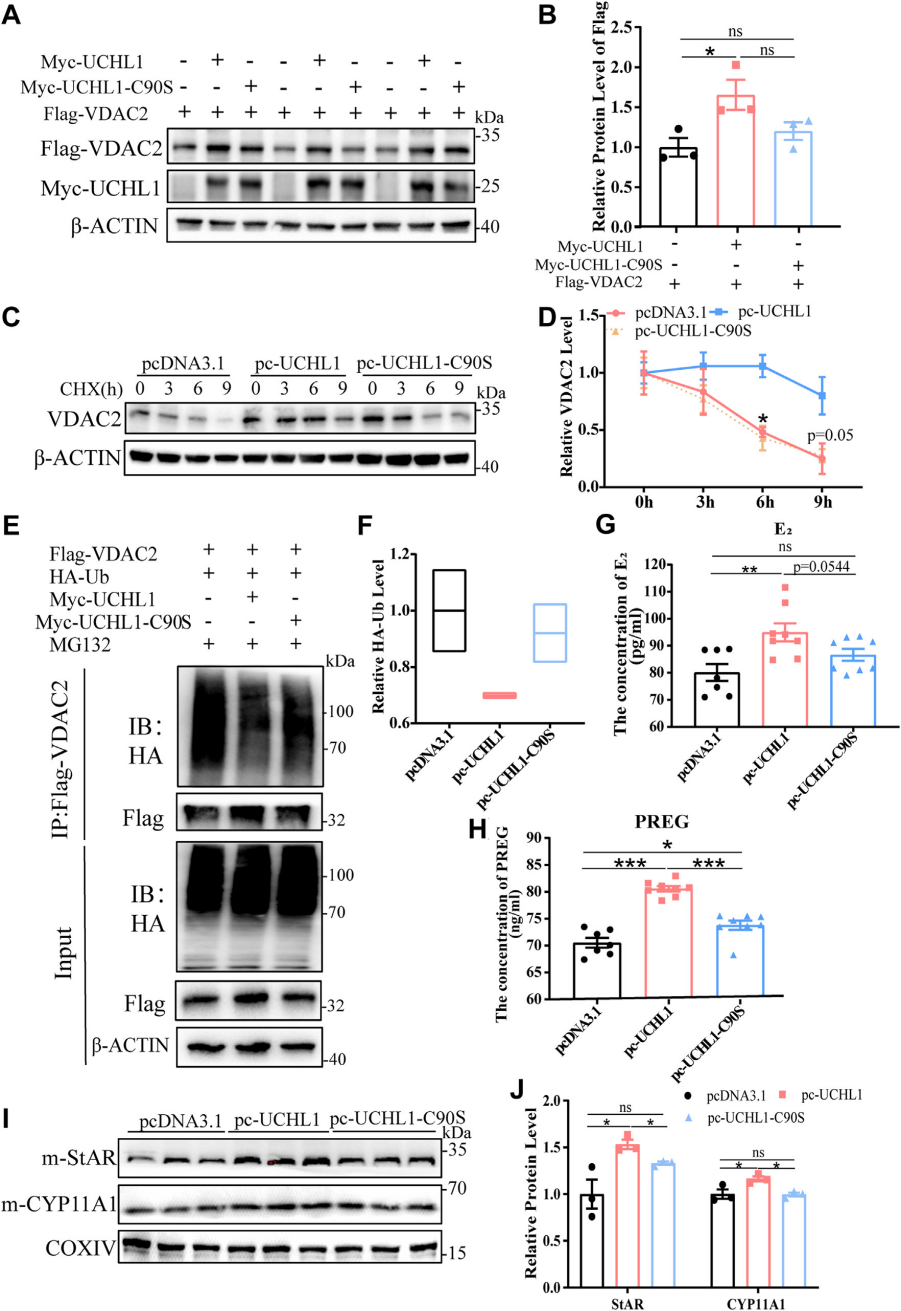
**C90 of UCHL1 is necessary for its deubiquitination activity.***A*, the protein levels of Flag-VDAC2 and Myc-UCHL1 after transfection with Myc-UCHL1 and Myc-UCHl1-C90s, respectively, under the overexpression of Flag-VDAC2. *B*, quantitative protein levels of Flag-VDAC2 under (*A*) treatments. *C*, GCs were overexpressed with UCHL1 or UCHL1-C90S and were treated with 25 μg/ml cycloheximide (CHX) for the indicated times. The whole cell lysates were assayed by using western blots. *D*, the quantitative protein level of VDAC2 under (*C*) treatment. *E*, GCs were transfected with a plasmid for Flag-VDAC2 and HA-Ub, with the empty vector or expression vector for Myc-UCHL1 (WT or C90S), and treated with MG132 (10 μM). Cell lysates were immunoprecipitated with anti-Flag and Western blot analysis with anti-Flag and anti-HA. *F*, quantitative protein levels of HA-Ub under (*E*) treatments. *G* and *H*, E_2_ (*G*) and PREG (*H*) concentrations were measured using ELISA. Culture supernatants were collected 24 h after pcDNA3.1, pc-UCHL1, and pc-UCHL1-C90S treatments. *I*, protein levels of StAR and CYP11A1 in GC mitochondria in different treatments. *J*, quantitative protein levels of StAR and CYP11A1. Data are means ± SEMs of three independent experiments; ∗*p* < 0.05, ∗∗*p* < 0.01, ∗∗∗*p* < 0.001. GC, granulosa cell; UCHL1, ubiquitin C-terminal hydrolase L1; VDAC2, voltage-dependent anion channel 2.

UCHL1 is a DUB whose enzymatic activity relies on the C90 site (13). This fact led us to speculate that C90-mutated UCHL1 would lose its stabilizing effect on VDAC2. We constructed Myc-UCHL1-C90S mutant and Myc-UCHL1-WT vectors to test this hypothesis and transfected them into GCs. The Myc-UCHL1-C90S mutant group weakened the stability of Myc-UCHL1-WT on VDAC2 (Fig. 7, *A* and *B*). Unlike the WT-UCHL1 group, the C90-mutant group did not enhance the half-life of VDAC2 (Fig. 7, *C* and *D*), which may be caused by the partial loss of deubiquitination of UCHL1-C90S to VDAC2. As expected, overexpression of UCHL1 (WT) significantly abrogated VDAC2 ubiquitination and increased VDAC2 levels, whereas this effect was reversed by C90S-UCHL1 (Fig. 7, *E* and *F*). These findings suggest that because UCHL1-C90S cannot stabilize VDAC2, it weakens the synergistic effect with StAR and CYP11A1 in mitochondria (Fig. 7, *I* and *J*), reduces the transport of cholesterol to mitochondria, and thus inhibits E_2_ and PREG compared with the WT-UCHL1 group (Fig. 7, *G* and *H*). These findings suggest that the C90 site of UCHL1 plays an essential role in deubiquitination enzyme activity during E_2_ synthesis in GCs.

### Lys45 and Lys64 in VDAC2 are essential for ubiquitination and degradation

UCHL1 deubiquitinates VDAC2 to stabilize its expression levels. The carboxyl group on the glycine of ubiquitin is an isopeptide bonded to the amino of the substrate protein lysine. There is a question as to which lysine site of VDAC2 is related to this modification. K45 and K64 sites of VDAC2 in the si-UCHL1 group were significantly upregulated compared with the control group, identified in the ubiquitinome.

**Figure 8:**
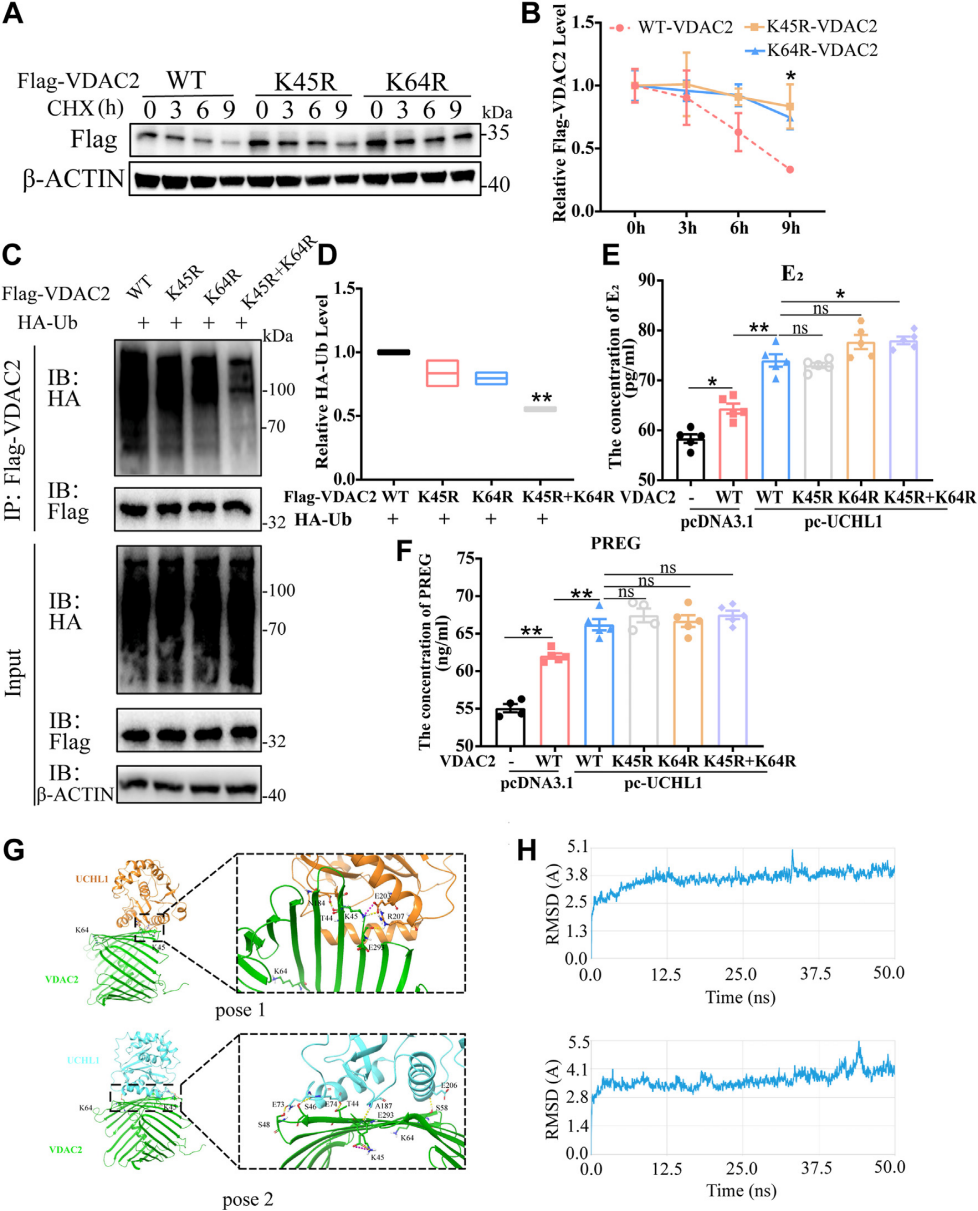
**The K45 and K64 sites are critical for VDAC2 degradation.***A*, GCs were transfected with Flag-VDAC2 (WT, K45R, or K64R) and treated with CHX (25 μg/ml) for the indicated times. The expression levels of Flag-VDAC2 were analyzed using Western blotting. *B*, quantification of the expression levels of WT, K45R, and K64R Flag-VDAC2. *C*, immunoprecipitation and Western blot analysis of GCs transfected with vectors expressing Flag-VDAC2 (WT, K45R, K64R, or K45R+K64R) and HA-Ub. *D*, quantification of the expression levels of HA-Ub. *E* and *F*, E_2_ (*E*) and PREG (*F*) concentrations were measured using ELISA. Culture supernatants collected 24 h after GCs were transfected with a plasmid for pcDNA3.1 and pc-UCHL1 with the expression vector for VDAC2 (WT, K45R, K64R, and K45R+K64R). *G*, schematic diagram of the two most likely molecular docking conformations where UCHL1 forms a hydrogen bond or salt bridge with K45 or K64 of VDAC2. *H*, RMSD of the protein–protein complex after 50 ns of molecular dynamics. Data are means ± SEMs of three independent experiments; ∗*p* < 0.05, ∗∗*p* < 0.01. CHX, cycloheximide; GC, granulosa cell; UCHL1, ubiquitin C-terminal hydrolase L1; VDAC2, voltage-dependent anion channel 2.

We created mutant constructs by substituting lysine residues with arginines (R) and found that UCHL1 failed to stabilize the K45R and K64R VDAC2 mutants, as did the WT (Fig. S3*A*). We used a CHX-chase assay and observed that the degradation rate of the K45R-VDAC2 and K64R-VDAC2 mutants was slower than WT-VDAC2 (Fig. 8, *A* and *B*). And the double mutants of (K45R+K64R)-VDAC2 has a longer half-life (Fig. S3, *B* and *C*). The ubiquitination assay also showed that the K45R-VDAC2 and K64R-VDAC2 mutants displayed reduced ubiquitination, and the double mutant of (K45R+K64R)-VDAC2 had the most significant effect (Fig. 8, *C* and *D*). This finding suggests that K45 and K64 are critical ubiquitination sites of VDAC2. While UCHL1 was overexpressed, WT-VDAC2 and K45R-VDAC2, K64R-VDAC2, (K45R+K64R)-VDAC2 showed no significant difference in E_2_ and PREG synthesis in GCs (Fig. 8, *E* and *F*). Because the critical lysine site of VDAC2 binding to the ubiquitin chain was mutated, its expression levels were stable.

Protein-protein docking experiments showed that UCHL1 protein formed hydrogen bonds or salt bridges with K45 and K64 of VDAC2 or its surrounding amino acid residues (Figs. 8*G* and S4, *A*–*C*), and pose1 and pose2 had higher PIPER pose scores and larger PIPER cluster sizes (Fig. S4*D*). After 50 ns of molecular dynamics, the molecular dynamics locus was analyzed, and the RMSD of locus proteins was extracted. The RMSD of the protein–protein complex remained relatively stable after 45 ns (Fig. 8*H*), and the binding free energies were −159.65 kcal/mol and −101.48 kcal/mol, respectively (Fig. S4*E*). These results suggest that UCHL1 removes the ubiquitin chains on K45 and K64 in VDAC2 to prevent it from degradation, thereby synergizing with StAR and CYP11A1 in mitochondria to promote cholesterol transport and ultimately enhance E_2_ synthesis.

### Inhibition of UCHL1 disrupted the estrous cycle and decreased E_2_ levels in female mice

**Figure 9:**
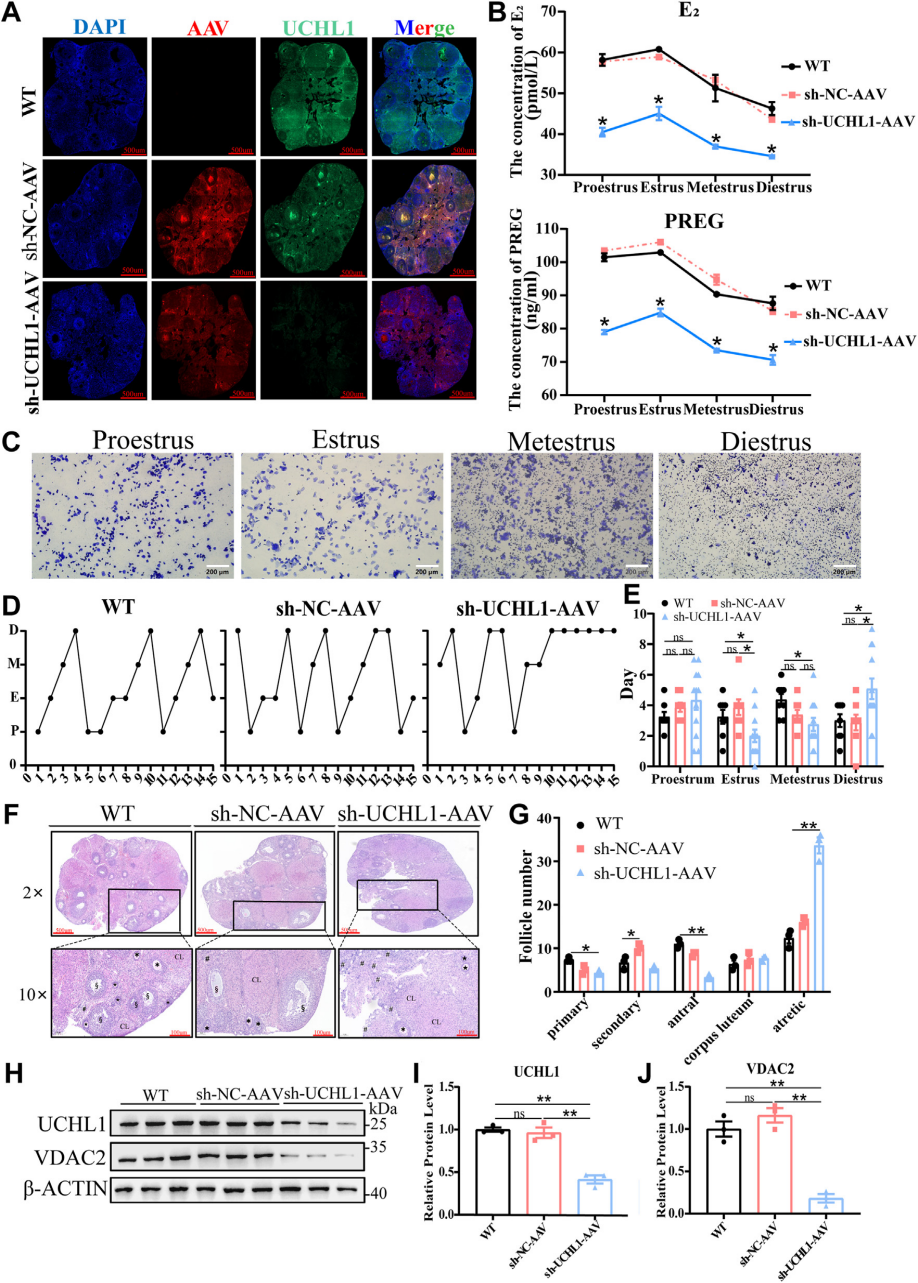
**The effects of UCHL1 inhibition on hormone secretion and follicular development in female mice.***A*, immunofluorescence analysis of WT and injected with sh-NC-AAV and sh-UCHL1-AAV mouse ovarian tissue (n = 3). Ovarian tissue was collected during the sixth week of *in situ* ovarian injection of AAV. AAV is labeled with mcherry red fluorescent, and *green* represents UCHL1. *B*, the serum of female mice was collected to determine the E_2_ and PREG levels (n = 8). *C*, representative image of vaginal smear staining to detect the estrus cycle; scale bar represents 200 μm. *D* and *E*, statistics of various stages of the estrous cycle (n = 8). *F*, representative images of H&E-stained sections of an ovary (n = 3); scale bars represent 500 μm and 100 μm. *G*, statistics of the follicle numbers during various developmental periods (n = 3), ^★^, primary follicle; ∗, secondary follicle; §, antral follicle; #, atretic follicle, CL, corpora lutea. *H*, protein levels of UCHL1 and VDAC2 in WT, sh-NC-AAV, and sh-UCHL1-AAV ovarian tissue (n = 5). *I* and *J*, quantitative protein levels of UCHL1 (*I*) and VDAC2 (*J*). Data are means ± SEMs of three independent experiments; ∗*p* < 0.05, ∗∗*p* < 0.01. UCHL1, ubiquitin C-terminal hydrolase L1; VDAC2, voltage-dependent anion channel 2.

To verify the effect of UCHL1 on reproductive performance in mice *in vivo*, sh-NC-AAV and sh-UCHL1-AAV were injected into mouse ovarian tissue. The immunofluorescence results of the ovarian tissue in the WT and sh-NC-AAV groups showed that UCHL1 fluorescence filled the entire ovarian tissue, but little UCHL1 fluorescence was observed in the sh-UCHL1-AAV group (Fig. 9*A*). This finding suggests that UCHL1 was knocked down in ovarian tissue. The hormone content of mice in the three groups peaked in estrus and was lowest in diestrus; serum E_2_ and PREG levels were significantly reduced after UCHL1 inhibition (Fig. 9*B*). In addition, compared with the control, the estrous cycle was disordered in the sh-UCHL1-AAV group, with a shortening of the days of estrus but longer diestrus periods (Fig. 9, *C*–*E*). In the sh-UCHL1-AAV group, primary and antral follicles were relatively small, whereas the numbers of atretic follicles were significantly higher (Fig. 9, *F* and *G*). UCHL1 knockdown decreased the expression of VDAC2 protein level (Fig. 9, *H*–*J*), also consistent with the results in porcine GCs. These findings suggest that UCHL1 inhibition significantly decreases E_2_ levels, estrus days, and numbers of antral follicles while it increases the numbers of atretic follicles.

## Discussion

UCHL1 is a well-known deubiquitinase with essential roles in regulating neurodegenerative diseases (22), cancer (23), and even cardiac function (13) by abrogating the ubiquitination of many target proteins. The balance of UCHL1 is essential for maintaining germ cell and tissue homeostasis (24). For example, UCHL1 promotes oocyte maturation, fertilization, and embryogenesis (25). UCHL1 is associated with endometrial development, and its deletion leads to infertility (26). In addition, a recent study showed that UCHL1 knockdown resulted in stunted ovarian development and decreased litter-size in mice (20), consistent with previous studies that identified UCHL1 as a candidate gene affecting litter sizes in Erhualian pigs (19). We found that UCHL1 is more highly expressed in the ovarian tissues of high-yielding Yorkshire × Landrace sows than in low-yielding sows. UCHL1 levels in ovarian tissue of Yorkshire estrous sows were higher than in nonestrous sows. These effects are associated with E_2_ synthesis of GCs; nevertheless, the role of UCHL1 in E_2_ synthesis and its direct substrates remain largely unknown. In this study, we found that UCHL1 promoted E_2_ synthesis, and the levels of StAR and CYP11A1 related to E_2_ synthesis were also increased (Fig. 2). StAR and CYP11A1 are responsible for converting free cholesterol into PREG, a precursor to E_2_ synthesis (27). Then, does UCHL1 promote E_2_ synthesis by regulating the target protein during PREG synthesis, which in turn regulates the expression of StAR and CYP11A1? This is worthy of further exploration to screen target proteins that UCHL1 may affect E_2_ synthesis.

Theoretically, a true proteolysis-associated substrate of UCHL1 would be degraded by UCHL1 interference through its elevated protein ubiquitination level and interact with UCHL1 (28). Therefore, we carried out the first comprehensive screening of the substrate proteins through which UCHL1 regulates the physiological functions of GCs. Our ubiquitinome analysis identified 353 proteins with upregulated ubiquitination levels in response to UCHL1 knockdown, suggesting that UCHL1 is involved in cholesterol metabolic pathways and steroid biosynthesis. We then identified 367 proteins from our interactome data using an IP-MS assay. These ubiquitination level–upregulated UCHL1-interacting proteins may be potential targets of UCHL1 deubiquitination but may also be indirect effects; for example, UCHL1 upregulates the ubiquitination level of indirectly binding proteins by affecting target proteins.

VDAC2 (a mitochondrial porin) is a family of β-barrel membrane proteins most abundant in the mitochondrial outer membrane (29). VDAC2 is related to cholesterol metabolism and steroidogenesis (30). Previous studies showed that the expression levels of VDAC2 in dominant bovine follicles are significantly higher than in subordinate follicles (31), suggesting that VDAC2 has a positive and direct relationship with E_2_ synthesis. Prasad and other researchers found that VDAC2 interacted with StAR *via* its C-terminal 20 amino acids and N-terminal amino acids 221 to 229, regulating the mitochondrial processing of StAR into the mature protein. In the absence of VDAC2, StAR could not enter the mitochondria and therefore steroidogenesis was inhibited (21). Posttranslational modifications play a critical role in regulating VDAC2 stability (32, 33) including its role as a ubiquitin ligase substrate in ferroptosis (32). Li *et al*. reported that in nasopharyngeal carcinoma tumor models, as a substrate of E3 ubiquitin ligase-TRIM21, VDAC2 is degraded by TRIM21 *via* ubiquitination of K48 ligand, thereby inhibiting type-I interferon responses (34). However, the effect of the deubiquitination enzyme on VDAC2 has not been reported. Our findings showed that VDAC2 is a potential target for UCHL1 through ubiquitinome and IP-MS. We provided evidence that UCHL1 is stably associated with VDAC2 and reduces the ubiquitination of VDAC2, leading to its longer half-life and the inhibition of its degradation in GCs. Furthermore, VDAC2 knockdown abolished the UCHL1 overexpression-mediated increase of E_2_ and PREG levels and the increased levels of StAR and CYP11A1 in mitochondria (Fig. 6), suggesting that VDAC2 stability and activity are essential for the beneficial effect of UCHL1 on E_2_ synthesis.

UCHL1 has a catalytic structure triplet composed of cysteine, aspartic acid, and histidine. C90 is directly involved in catalysis, probably as the active site nucleophile; when mutated to serine, the deubiquitinase activity is declined by 10^7^ (35). To clarify the role of the C90 site of UCHL1 in regulating deubiquitination in GCs, we constructed the Myc-UCHL1-C90S mutant vector. The effects of UCHL1 on inhibiting the ubiquitination level of VDAC2 and prolonging the half-life of VDAC2 were significantly weakened by deubiquitinase-inactive variant C90S, and E_2_ synthesis and PREG levels in the deubiquitinase inactive variant (UCHL1-C90S) group significantly decreased, and expression levels of StAR and CYP11A1 in mitochondria were markedly decreased, respectively, with almost no difference from the control group (Fig. 7). These findings highlight the importance of the C90 site for deubiquitinase activity and are consistent with previous studies in which a UCHL1-C90S mutant significantly reduced the deubiquitination of target proteins (36).

In contrast, ubiquitination at sensitive sites destabilizes the native structure and increases the rate of proteasomal degradation (37), and ubiquitination modifications usually happen on lysine residues of target proteins (38). Lysine 45 and 64 are critical sites for ubiquitination and degradation of VDAC2, while UCHL1 reverses this process. When the K45 and K64 sites of VDAC2 were mutated, the ubiquitination level of VDAC2 was decreased and its stability was enhanced, while UCHL1 could not enhance the PREG and E_2_ synthesis levels in the mutant group (Fig. 8); this finding is because VDAC2 has other sites that bind ubiquitin molecules but cannot be recognized explicitly by UCHL1, also suggested by the ubiquitinome results. The K120, K277, and K285 sites of VDAC2 were all recruited by ubiquitination antibodies; however, UCHL1 knockdown did not cause ubiquitination changes.

Our findings suggest a novel function of UCHL1 in GC E_2_ synthesis. First, UCHL1 overexpression promoted E_2_ synthesis, while inhibition of UCHL1 weakened E_2_ synthesis. Second, ubiquitinome analysis in response to UCHL1 knockdown suggested a role for UCHL1 in cholesterol metabolism and steroid synthesis. Third, the interactome identified VDAC2 as a protein that interacts with UCHL1. However, a limitation of our study is the absence of ovarian GC-specific UCHL1 KO mice. Woodman *et al*. reported that mice with systemic UCHL1 knockout showed significant disturbances of the estrus cycle (20); however, UCHL1 is expressed in gonadotropes of the pituitary gland (39). These effects are difficult to differentiate from ovarian effects, as the hypothalamic–pituitary–ovarian axis is complex and inter-dependent. Therefore, sh-UCHL1-AAV was injected into mouse ovarian tissue to simulate the effect of ovarian tissue-specific knockout. Mouse models were used to explore the effects of UCHL1 knockdown on ovarian function *in vivo*, including hormone secretion, estrous cycle, and follicle development.

The sh-NC-AAV group was studied to exclude the effect of surgical operation on the ovarian function of mice. We found that hormone levels, the estrous cycle, and follicle development of mice in the sh-NC-AAV group were not different from those of the WT group. Furthermore, serum hormone levels peaked during estrus and were lowest during diestrus, consistent with normal estrus rules (40). However, after UCHL1 knockdown, the period in estrus was significantly reduced, the period in diestrus was significantly prolonged, and the number of atretic follicles was increased. The hypothalamic–pituitary–ovarian axis is a system in which pituitary gonadotrophins stimulate ovarian folliculogenesis and the production of steroid hormones, which in turn exercise feedback control on the production of pituitary gonadotrophins (41). Therefore, we speculated that E_2_ levels remained low in the sh-UCHL1-AAV group, which may not cause the positive feedback regulation of follicle-stimulating hormone. Moreover, low E_2_ levels did not inhibit apoptosis (42), thereby increasing the number of atretic follicles (43) and impairing follicle development.

**Figure 10:**
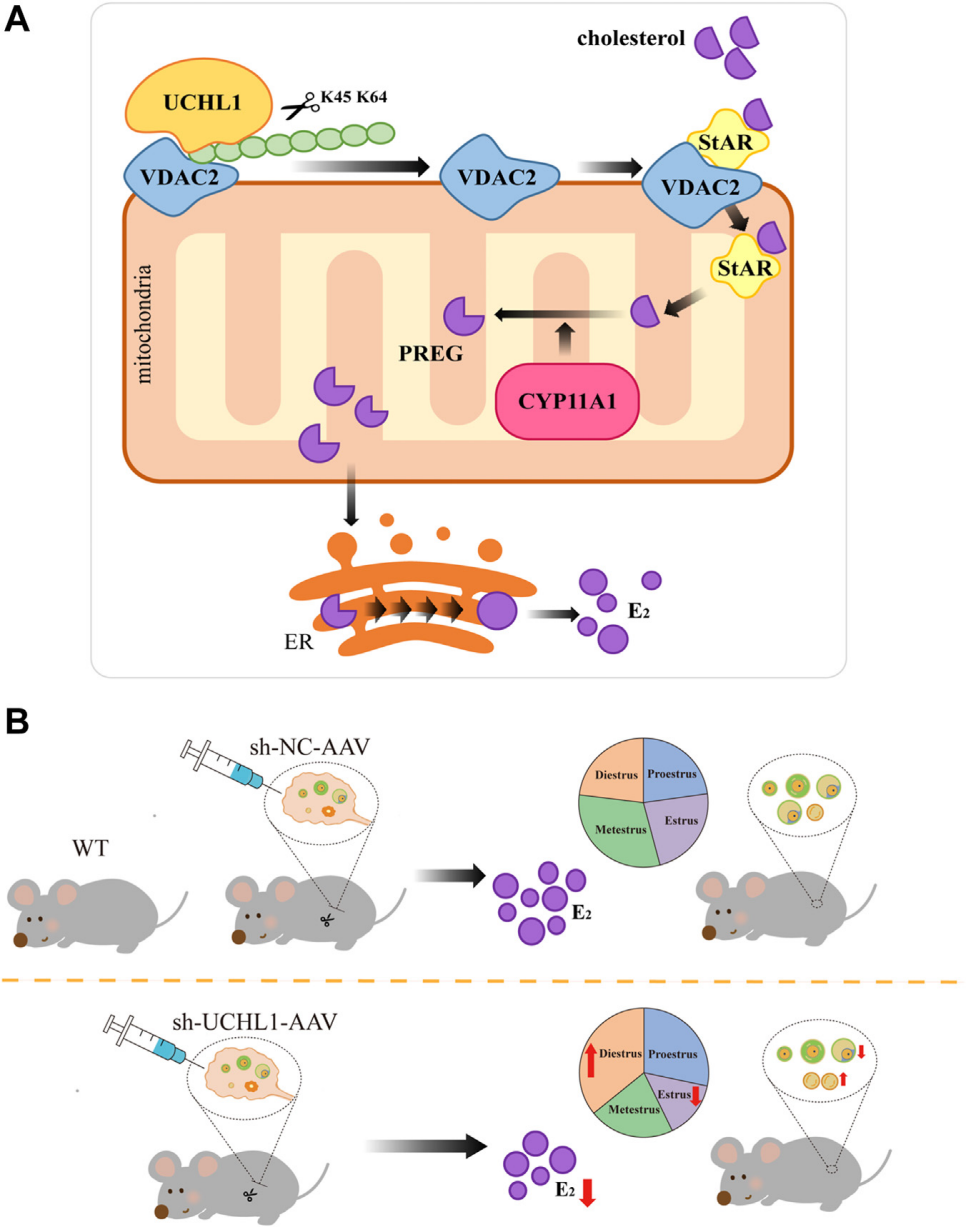
**Schematic summary of UCHL1 regulation of E**_**2**_**synthesis and follicle development.***A*, UCHL1 removes the ubiquitin chains at the K45 and K64 sites of VDAC2 to protect VDAC2 from degradation, thereby accelerating StAR-carrying cholesterol into mitochondria and ultimately promoting E_2_ synthesis. *B*, in *in vivo* experiments, UCHL1 knockdown resulted in decreased E_2_ levels, disrupted estrus cycles, reduced dominant follicle number, and increased atretic follicle number in mice. UCHL1, ubiquitin C-terminal hydrolase L1; VDAC2, voltage-dependent anion channel 2.

These findings suggest UCHL1 is a DUB that cleaves ubiquitin chains of VDAC2 at K45 and K64 and protects it from degradation through the proteasome pathway. Through VDAC2 stabilization, UCHL1 facilitates cholesterol transport to mitochondria and increases the expression of StAR and CYP11A1 proteins, thereby promoting E_2_ synthesis (Fig. 10*A*). In *in vivo* experiments, UCHL1 knockdown resulted in decreased E_2_ levels, disrupted estrus cycles, reduced dominant follicle number, and increased atretic follicle number in mice (Fig. 10*B*). These findings suggest that UCHL1 is a potential target for follicular development.

The similarities between pigs and humans regarding anatomy, physiology, and the genome enhance a pig’s potential as a human model (44). Moreover, the protein sequence of UCHL1 is highly conserved across species (Fig. S5). Our findings may serve as the basis for future clinical studies on human ovarian GCs and provide gene targets for screening treatment of inadequate human E_2_ secretion and follicle development.

## Experimental procedures

### Identification and collection of ovarian tissue from estrus and nonestrus gilts

Six hundred forty-three New French Large White sows with clear pedigree, good health, an average total litter of more than 12.5 piglets in the last three litters, and seven or more pairs of papillae from Wanrong Wenshi Animal Husbandry Co, Ltd were selected as the maternal parent to form the core group C0 generation and carry out pure propagation production. Thirty New French Large White gilts with two stable estrus periods in C1 generation were selected as the estrus group, and 30 full sibling gilts without estrus were selected as the nonestrus group. Six normal sows with two stable estrous periods and six nonestrus abnormal sows were selected for slaughter, and ovarian tissues were removed. The ovaries were used for subsequent detection of UCHL1 expression. All animal protocols were approved by the Committee for the Ethics on Animal Care and Experiments of Northwest Agriculture and Forestry University (No. NWAFU-202106025).

### Identification and collection of ovarian tissue from high- and low-yielding sows

We collected and collated litter-size records (8657 parity) from Hanshiwei Food Ltd, Co from 2016 to 2018 and used SPSS25.0 to perform normal distribution processing on the data. After normal transformation and testing, the total litter size (12.9 ± 2.17) was found to be approximately normally distributed, with a critical value of 15% right tail probability (14.7 head/litter) and a critical value of 15% left tail probability (9.3 head/litter). Therefore, we defined smaller litter sizes as groups with less than 9.3 head/litter and larger litter sizes as groups with greater than 14.7 head/litter. They were used for the subsequent detection of UCHL1 expression.

### Immunohistochemical staining

Ovaries were fixed overnight in 4% paraformaldehyde, embedded in paraffin, deparaffinized, rehydrated, and incubated with primary and secondary antibodies. Paraffin embedding, sectioning, and staining were performed at Yike Biotechnology Service Co, Ltd. These criteria were used to measure UCHL1 expression in GCs at various follicle stages.

### Porcine ovarian GC cultures and transfection

Experimental procedures were conducted following the Animal (Scientific Procedures) Act of China. Healthy ovaries were isolated, immediately placed into 37 °C normal saline, and transported back to the laboratory within 1 hour. Healthy follicles with 3 to 5 mm diameters were selected from the ovaries, and the follicular fluid was removed using a 10-mL syringe. Dulbecco’s modified Eagle’s medium/F12 (Gibco) medium containing 10% fetal bovine serum (Gibco) and 100 IU/ml penicillin and 100 μg/ml streptomycin were added to suspend the cells; the medium was gently beaten repeatedly until the cells were dispersed entirely. In each experiment, ovaries obtained from ten sows were used, and 20 ovaries were collected to obtain pooled GCs. These GCs were divided into control and experimental groups, inoculated into culture plates, and cultured in a 5% CO_2_ incubator at 37 °C. The inoculation density of GCs was 10^5^ cells/cm^2^.

After 24 h of cell-adherent growth, the cells were washed with PBS. pcDNA3.1-Myc-UCHL1 (G0217535-4), pcDNA3.1-Flag-VDAC2 (G0217535-2), pcDNA3.1-HA-Ub (G0217535-1) (General Biosystems), pcDNA3.1-Myc-UCHL1-C90S (XA0026844-8), pcDNA3.1-Flag-VDAC2-K45R (XA0026844-9), pcDNA3.1-Flag-VDAC2-K64R (XA0026844-10), pcDNA3.1-Flag-VDAC2-(K45R+K64R) (XA0026844-11) (Tsingke Biotechnology Co, Ltd) vectors were transfected respectively. si-UCHL1 (GenePharma) and plko.1-copGFP-2A-PURO vector that induces the knockdown of VDAC2 (Tsingke Biotechnology Co, Ltd) using X-tremeGENE HP DNA transfection reagent (Roche) when the cell density was approximately 50%. The sequences of the siRNA and shRNA used are shown in Table S1.

### Quantitative real-time PCR

Total RNA was extracted using TRIzol reagent (TaKaRa). mRNA was reverse-transcribed using a kit (TaKaRa), and the complementary DNA was analyzed using SYBR Green PCR mix (Vazyme) in an Applied Biosystems StepOnePlus system (Thermo Fisher Scientific). Relative gene expression was calculated using the 2^−ΔΔCt^ method. Gene expression was normalized to β-actin. The sequences of the primers used are shown in Table S2.

### Western blot analysis

Total protein was extracted using radioimmunoprecipitation assay buffer (Beyotime) supplemented with protease inhibitors (Pierce) at 4 °C. We scraped the lysed cells and centrifuged (rpm) at 4 °C for 10 min. Supernatant protein concentrations were determined using a BCA protein assay kit (Cwbio). A one-fourth volume of 5× loading buffer (Cwbio) was added to an aliquot of the supernatant, and 20 μg of protein was separated using SDS-PAGE and transferred to polyvinylidene fluoride membranes (Millipore). Membranes were incubated overnight with the following appropriate primary antibodies: anti-UCHL1 (AF5490, 25 kDa, Affinity Biosciences, 1:1000) and (sc-271639, 25 kDa), anti-VDAC2 (AF5397, 32 kDa, Affinity Biosciences, 1:1000), anti-COXIV (D262690, 20 kDa, Sangon Biotech), anti-HA (66006-2-Ig, Proteintech, 1:3000), anti-DYKDDDDK (20543-1-AP, Proteintech, 1:3000), anti-Myc (60003-2-Ig, Proteintech, 1:3000), anti-StAR (CY7082, 32 kDa, Abways, 1:1000), anti-CYP11A1 (DF4697, 60 kDa, Affinity Biosciences, 1:1000), or anti-CYP19A1 (DF3564, 58 kDa, Affinity Biosciences, 1:1000). The following day, the membranes were washed three times with Tris-buffered saline containing Tween 20. Then, an appropriate secondary antibody (Boster) was added, and the samples were incubated for 1 h at 4 °C with shaking. The secondary antibodies were horseradish peroxidase–conjugated goat anti-mouse IgG, goat anti-rabbit IgG, and rabbit anti-goat IgG. Finally, the signals were detected using a gel imaging system (Bio-Rad) and analyzed using Image J software (http://imagej.nih.gov/ij/).

### Enzyme-linked immunosorbent assay

E_2_ and PREG in the follicular fluid and medium were measured using a porcine E_2_ ELISA Kit (YJ002366, Mlbio) and PREG ELISA Kit (YJ911258, Mlbio) according to the manufacturer’s instructions. Briefly, the culture medium samples were centrifuged at 2000*g* for 20 min. The supernatants resolved in the dilution buffer were added to micro-ELISA strip plates precoated with an antibody specific to E_2_ or PREG, followed by 30 min incubation at 37 °C with a horseradish peroxidase–conjugated antibody against E_2_ or PREG. After washing, the substrate solution was added to trigger the chromogenic reaction. The absorbance was measured at 450 nm using a Multiskan TM FC spectrophotometer (Thermo Fisher Scientific). The ELISA kit was coated with monoclonal antibodies with no cross-reactions.

### Ubiquitinated peptide enrichment

GCs transfected with si-NC and si-UCHL1 for 24 h were collected, some of which were used to detect the interference efficiency of UCHL1; the remainder was used for ubiquitinome. Three biological replicates were carried out for ubiquitinome analysis. The samples were added to a lysis buffer (8 M urea, 1% protease inhibitor, 50 μM PR-619). After centrifugation at 4 °C at 12,000*g* for 10 min, cell debris was removed, and the supernatants were transferred to a new centrifuge tube for protein concentration determination using the BCA kit. Pancreatic enzymatic hydrolysis, ubiquitination modification enrichment, and LC-MS analysis were processed at PTM Biolabs. The enrichment method of ubiquitination peptides was described previously (45).

### Liquid chromatography tandem mass spectrometry analysis

Liquid chromatography tandem mass spectrometry (LC-MS/MS) analysis was completed in PTM Biolabs. The peptides were dissolved in liquid chromatography mobile phase A and separated using the EASY-nLC 1000 ultra-high performance liquid system. Mobile phase A was an aqueous solution containing 0.1% formic acid and 2% acetonitrile. Mobile phase B was an aqueous solution containing 0.1% formic acid and 90% acetonitrile. Liquid phase gradient settings were 0 to 40 min, 7%–25% B; 40 to 52 min, 25%–35% B; 52 to 56 min, 35%–80% B; 56 to 60 min, 80% B; the flow rate maintained at 500 nl/min. The peptides were separated on an ultra-high-performance liquid phase system and injected into an NSI ion source for ionization and analysis using Q Exactive Plus mass spectrometry. The ion source voltage was set to 2.1 kV, and the peptide's parent ions and secondary fragments were measured and analyzed using high-resolution Orbitrap. The scanning range of primary mass spectrometry was 350 to 1800 m/z, and the scanning resolution was 70,000.

### Immunoprecipitation-mass spectrometry

GCs were collected in three large dishes and lysed with IP cell lysate according to the manufacturer’s instructions (C500035, Sangon Biotech). Cell debris was removed by centrifugation at 13,000 rpm for 10 min, and the protein supernatants were incubated with magnetic beads (10003D, Thermo Fisher Scientific) for 1 h for preclearance. Then, the supernatants were subjected to IP with 10 μl anti-Myc overnight at 4 °C. On the following day, the protein supernatants of the incubated primary antibody were incubated with the magnetic beads for 2 h. The magnetic beads were cleaned three times with the lysate. The magnetic bead–protein complex was boiled in a 5 × loading buffer water bath for 15 min. The collected strips were subjected to enzymolysis and LC-MS/MS analysis, as described in the ‘Liquid chromatography tandem mass spectrometry analysis' section.

### Co-immunoprecipitation

Some of the lysis protein of GCs was reserved as input, half of the remaining protein was used to incubate IgG antibody and magnetic bead as a negative control, and the other half was incubated with UCHL1 or VDAC2 antibody and magnetic bead. UCHL1 pull-down protein was incubated with UCHL1 and VDAC2 antibodies by Western blot to determine whether UCHL1 would bind VDAC2. Similarly, proteins in the VDAC2 pull-down were incubated with UCHL1 and VDAC2 antibodies to determine whether VDAC2 would bind to UCHL1.

### *In vivo* ubiquitylation assays

To assess endogenous VDAC2 ubiquitination, GCs were transfected with siRNA-control or si-UCHL1 for 24 h. VDAC2 was immunoprecipitated with anti-VDAC2 and detected by Western blot analysis with an antibody against ubiquitin (Ub). To evaluate the effect of UCHL1-C90 mutants and K45-VDAC2, K64-VDAC2, (K45+K64)-VDAC2 mutants on the ubiquitination level of VDAC2, GCs were transfected with expression vectors for Flag-VDAC2 (WT or K45R, K64R, K45R+K64R mutant), HA-Ub, Myc-UCHL1 (WT or C90S mutant). Lysate proteins from cultured cells were precipitated and analyzed by Western blotting using appropriate antibodies (*i.e.*, VDAC2, Ub, HA, Flag, and Myc).

### Fluorescence microscopy

GCs were washed with PBS and postfixed in 4% paraformaldehyde for 15 min. After the cells were blocked in PBS containing 1% bovine serum albumin and 0.2% Triton X-100 for 10 min, monoclonal antibody against VDAC2 and UCHL1 was added at 1:200 for overnight incubation at 4 °C. After washing with PBS three times for 5 min, the cells were then incubated with secondary antibody for 1 h, and the nuclear staining was performed with 4′,6-diamidino-2-phenylindole (100 ng/ml) for 5 min. The stained cells are photographed on a Spinning Disk Confocal Microscope (2017165203).

### Extraction of mitochondrial proteins

Mitochondrial proteins were extracted from GCs after different transfection treatments using a kit (KGP850, KeyGEN BioTECH). GCs were lysed in precooled lysis buffer 1, centrifuged at 800*g* for 5 min to remove debris and large organelles, and centrifuged at 15,000*g* for 10 min to obtain mitochondria. Mitochondrial proteins are lysed in lysis buffer 2.

### Animal samples and microinjection into ovaries

Eight-week-old Institute of Cancer Research female mice were purchased from the Xi’an Branch of Chongqing Tengxin Biotechnology Co, LTD, and qualified by the Laboratory Animal Center of Xi’an Jiaotong University Health Science Center. The animals were housed in temperature- and light-controlled conditions (22–24 °C, 12-h light-dark cycle) with ad libitum access to water and food. After a week of adaptation, mice (n = 30) were randomly divided into WT (n = 8), sh-NC-AAV (n = 10), and sh-UCHL1-AAV (n = 12) groups.

Two dorsal incisions were made approximately 2 mm caudal to the last rib for the ovarian injections. The ovary was removed using fine forceps. Care was taken to avoid injuring blood vessels and the ovarian tunica. A pipette was advanced with the micromanipulator by gently holding a fat pad around the oviduct and ovarian bursa, and a glass needle was inserted under the tunica albuginea of the ovary. Approximately, 10 μl of mCherry-sh-UCHL1-NC/AAV (1 × 10^12^ V g/ml; HANBIO) was microinjected into the ovarian stroma. The endothelium and outer skin were carefully sutured, and the mice were placed in a warm space for recovery. The mice were awake and returned to voluntary activity within half an hour after surgery.

### Vaginal cytology

A vaginal swab sample was collected using a pipette tip aspirated with 10 μl ambient temperature physiological saline and inserted into the vagina of the restrained mouse. Blowing gently several times, the cells were transferred to a dry glass slide. The slide was air-dried and stained with approximately 400 μl 0.1% crystal violet (Sigma-Aldrich) for 1 min. The slides were rinsed with water for 1 min, dried in the air, and viewed immediately at 10 × magnification under bright field illumination. Proestrus was defined as several nucleated epithelial cells and a few keratinized epithelial cells. Estrus was defined as a large number of keratinized epithelial cells and a small amount of nucleated epithelial cells. Metestrus was defined as the presence of keratinized epithelial cells and white blood cells. Diestrus was defined as the presence of almost all white blood cells (46).

### H&E staining

Mouse ovarian tissue was removed intactly and fixed in 4% paraformaldehyde. The fixed ovaries were dehydrated and processed for paraffin embedding, and 5-μm sections underwent H&E staining. Dewaxing, hydration, hematoxylin staining, differentiation and antibluing, eosin staining and dehydration, and air-dry sealing were performed on the H&E-stained samples.

The number of follicles in each period was statistically analyzed on H&E-stained samples. The primary follicle was characterized by a cuboidal layer of GCs around an oocyte. The number of GC layers in secondary follicles increased, there was sizable antral space in the antral follicles, and the volume of the follicles increased significantly. Atretic follicles were defined as shrinking and absent oocytes, and GCs were replaced by fibrous material.

### Protein-protein docking, molecular dynamics simulation, and binding free energy analysis

The crystal structure of UCHL1 and VDAC2 proteins was searched in the PDB (https://www.rcsb.org/). For VDAC2, the 7NIE cryo-electron microscopic crystal structure of porcine and the SWISS-MODEL homologous modeling platform (https://swissmodel.expasy.org/) was used to predict the UCHL1 protein sequence of three-dimensional protein structures. The Protein Preparation Wizard module of Schrodinger software (https://www.schrodinger.com) was used for protein preparation. The protein-protein docking (Piper) module was used to perform molecular docking. We selected five conformations in which UCHL1 formed hydrogen bonds or salt bridges with K45 and K64 of VDAC2 or acted with residues around them. The Desmond module in the Schrodinger drug design package simulated the docking conformation. After 50 ns molecular dynamics simulation, the RMSD of pose1 and pose2 was calculated, and hydrogen bonds formed by UCHL1 to K45 and K64 of VDAC2 were analyzed. Finally, molecular mechanics-generalized born surface area was used to calculate the binding free energy.

### Bioinformatic analysis

Functional enrichment analysis was performed using the UniProt-GOA database (http://www.ebi.ac.uk/GOA/), InterPro (http://www.ebi.ac.uk/interpro/), and Kyoto Encyclopedia of Genes and Genomes annotation using two-tailed Fisher’s exact tests. The pathway with a corrected *p*-value < 0.05 was considered significant. Amino acid sequences of different species were obtained from NCBI (http://www.ncbi.nlm.nih.gov), including *Sus scrofa*, *Homo sapiens*, *Rattus norvegicus*, *Mus musculus*, *Bos taurus*, *Moschus berezovskii*, *Nyctereutes procyonoides*, *Pan panis*cus, *Ovis aries*, *Capra hircus*, *Equus caballus*, *Oryctolagus cuniculus*, and *Felis catus*. The homology of amino acid sequences was compared using MegAlign software (https://www.dnastar.com/).

### Statistical analysis

Data were analyzed using GraphPad Prism 7.0 (https://www.graphpad-prism.cn/), and all data were expressed as means ± SEMs of three independent experiments. Data were analyzed using the Student’s *t* test, one-way, or two-way ANOVA. Values were considered significant for *p*-values less than 0.05 (∗*p* < 0.05; ∗∗*p* < 0.01; ∗∗∗*p* < 0.001).

## Data availability

The data that support the findings of this study are available from the corresponding author upon reasonable request.

## Supporting information

This article contains supporting information (Figs. S1–S5; Tables S1 and S2; Supplementary Datas S1 and S2).

## Conflict of interest

The authors declare that they have no conflicts of interests with the contents of this article.
