# Supplementary material for: Deubiquitinase UCHL1 regulates estradiol synthesis by stabilizing voltage-dependent anion channel 2
Source: J Biol Chem. 2023 Oct 4;299(11):105316. doi: 10.1016/j.jbc.2023.105316 (PMC10656229; doi:10.1016/j.jbc.2023.105316)
Supplement: Supporting information [file mmc1.docx]

Supporting Information

**Deubiquitinase UCHL1 regulates estradiol synthesis by stabilizing** **voltage-dependent anion channel 2**

Shengjie Shi^1,2†^, Guiyan Chu^1,2†^, Lutong Zhang^1,2^, Huan Yuan^1,2^, Mielie Madaniyati^1,2^, Xiaoge Zhou^1,2^, Liguang Wang^1,2^, Chuanjiang Cai^1^, Weijun Pang^1,2^, Lei Gao^1,2^, Gongshe Yang^1,2^*

1 College of Animal Science and Technology, Northwest A&F University, Yangling 712100, China

2 Key Laboratory of Animal Genetics, Breeding and Reproduction of Shaanxi Province, Yangling 712100, China

* Correspondence: gsyang@nwafu.edu.cn

† These authors have contributed equally to this work

**Contents of supporting information**

**Supplementary Fig. 1-5 and Supplementary Table 1 and Table 2**


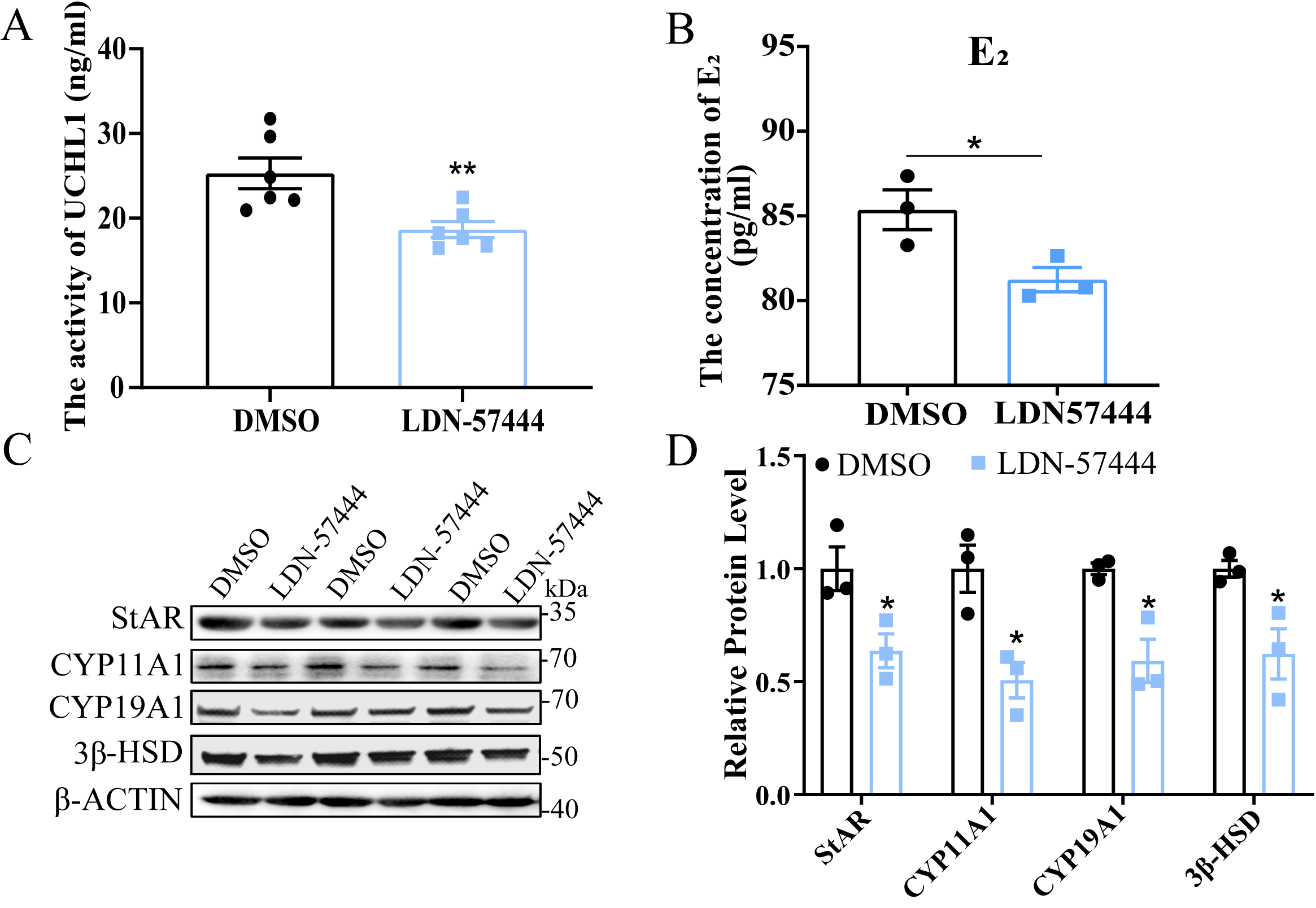


**Fig. S1. LDN57444 inhibits E_2_ synthesis.** (A) The activity of UCHL1 after 3-h treatment with 1 μmol/L LDN-57444. (B) E_2_ concentration was measured using ELISA after treatment with LDN-57444. (C) Western blot analysis of critical proteins in E_2_ synthesis. (D) Quantification of the western blot analysis. Data are means ± SEMs of three independent experiments; **P* < 0.05, ***P* < 0.01.

**Fig. S2. Protein motif analysis and domain prediction of ubiquitination upregulation sites.** (A) Heat map of amino acid frequency variation near modification sites based on MOMO analysis. (B) Protein domain prediction of upregulation of ubiquitination sites.


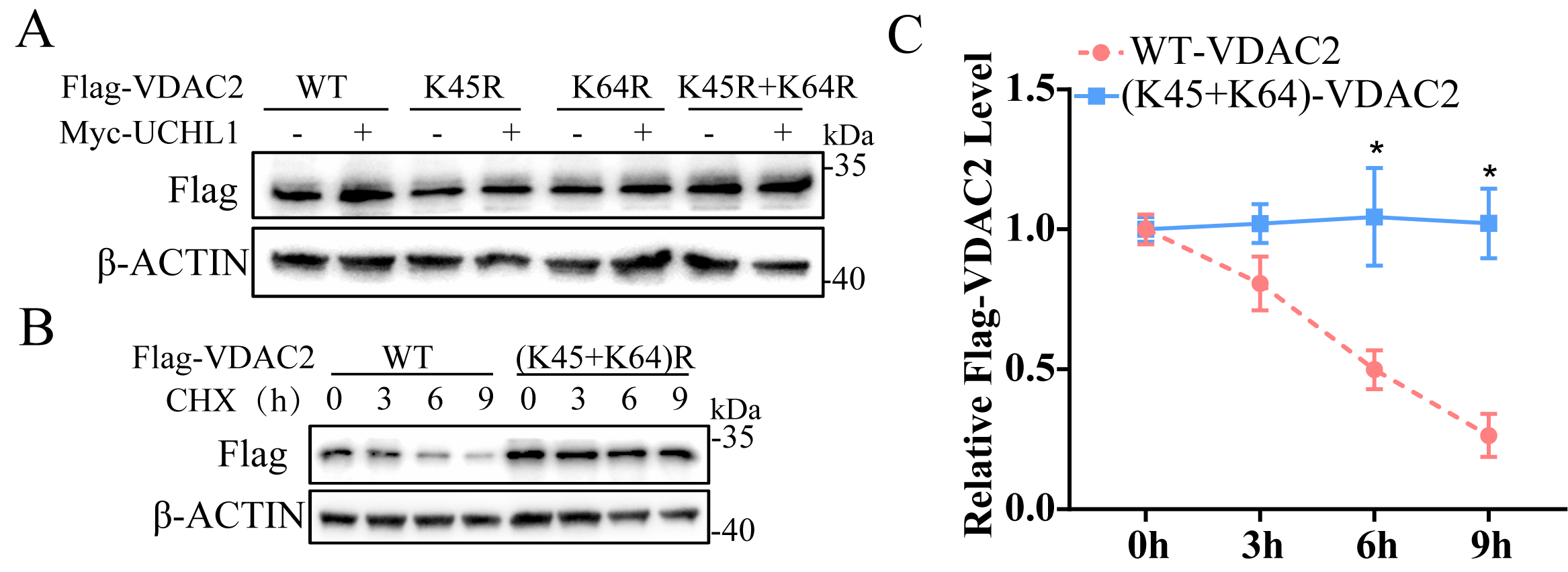


**Fig. S3. Mutations at K45 and K64 of VDAC2 inhibit its degradation.** (A) Western blot analysis of protein extracts of GCs transfected with empty vector or vector for Myc-UCHL1 with a plasmid expressing WT, K45R, K64R, or K45R+K64R mutant of Flag-VDAC2. (B) GCs were transfected with Flag-VDAC2 (WT, K45R+K64R) and treated with CHX (25 µg/mL) for the indicated times. Expression levels of Flag-VDAC2 were analyzed using western blotting. (C) Quantification of the expression levels of WT, K45R+K64R mutant of Flag-VDAC2. Data are means ± SEMs of three independent experiments; **P* < 0.05.

**Fig. S4. Conformation and molecular dynamics analysis of UCHL1 binding to K45 and K64 of VDAC2.** (D) Schematic diagram of the other three molecular docking conformations where UCHL1 forms a hydrogen bond or salt bridge with K45 or K64 of VDAC2. (E) PIPER pose scores and cluster sizes for five conformations. (F) The binding free energy of the last frame (50 ns) in equilibrium state after molecular dynamics simulation of pose1 and pose2.

**Fig. S5. Homology analysis of the amino acid sequence of UCHL1 among species.**

**Supplementary Tables**

**Table S1. siRNA and shRNA sequences (pig).**

| Gene | Sequence | |
| --- | --- | --- |
|  | Forward Sequence | Reverse Sequence |
| si-NC | UUCUCCGAACGUGUCACGUTT | ACGUGACACGUUCGGAGAATT |
| si-UCHL1 | GUCCUAAGGUGUACUUUAUTT | AUAAAGUACACCUUAGGACTT |
| sh-NC | CCGG-GGACTTGCTGCTGCACGAA-CTCGAG-TTCGTGCAGCAGCAAGTCC-TTTTTG | AATT-CAAAAA-GGACTTGCTGCTGCACGAA-CTCGAG-TTCGTGCAGCAGCAAGTCC |
| sh-VDAC2-1 | CCGG-AGTCAACAACTCTAGTTTA-CTCGAG-TAAACTAGAGTTGTTGACT-TTTTTT | AATT-AAAAAA-AGTCAACAACTCTAGTTTA-CTCGAG-TAAACTAGAGTTGTTGACT |
| sh-VDAC2-2 | CCGG-GGTTCATCTAATACAGACA-CTCGAG-TGTCTGTATTAGATGAACC-TTTTTT | AATT-AAAAAA-GGTTCATCTAATACAGACA-CTCGAG-TGTCTGTATTAGATGAACC |
| sh-VDAC2-3 | CCGG-GCTAAATCAAAGCTGACAA-CTCGAG-TTGTCAGCTTTGATTTAGC-TTTTTT | AATT-AAAAAA-GCTAAATCAAAGCTGACAA-CTCGAG-TTGTCAGCTTTGATTTAGC |

**Table S2. Primers used in real-time quantitative PCR (pig).**

| Gene | Primer Sequence | |
| --- | --- | --- |
|  | Forward Primer | Reverse Primer |
| sus-Star | CGTTTAAGCTGTGTGCTGGG | TCCATGACCCTGAGGTTGGA |
| sus-Cyp11a1 | GGGCAACCCATTTCCTACCA | CGAGCACTGGTGGTACAGAC |
| sus-Cyp19a1 | TCCGCAATGACTTGGGCTAC | GCCTTTTCGTCCAGTGGGAT |
| sus-3β-HSD | TCTTGTCTGCTTCTCGCCAC | CAACTGAGACTTGGGTGCCA |
| sus-UCHL1 | ACTGGAGGAGGAGTCTTTGGG | TTCTTGTCCCTTCAGCTCTTCA |
| sus-VDAC2 | TGGTTCATCTAATACAGACACTGG | ACAAATCTGGTCTTCAATAGCG |
| sus-β-actin | GTCCCTGACCCTCCCAAAAG | GCTGCCTCAACACCTCAACCC |
